# Integration of omics studies indicates that species-dependent molecular mechanisms govern male fertility

**DOI:** 10.1186/s40104-023-00836-1

**Published:** 2023-03-02

**Authors:** Yoo-Jin Park, Won-Ki Pang, Myung-Geol Pang

**Affiliations:** Department of Animal Science & Technology and BET Research Institute, Chung-Ang University, Anseong, Gyeonggi-do, 17546 Republic of Korea

**Keywords:** Integrated signaling pathways, Male fertility, Metabolomics, Proteomics, Spermatozoa transcriptomics

## Abstract

**Background:**

Comparative and comprehensive omics studies have recently been conducted to provide a comprehensive understanding of the biological mechanisms underlying infertility. However, because these huge omics datasets often contain irrelevant information, editing strategies for summarizing and filtering the data are necessary prerequisite steps for identifying biomarkers of male fertility. Here, we attempted to integrate omics data from spermatozoa with normal and below-normal fertility from boars and bulls, including transcriptomic, proteomic, and metabolomic data. Pathway enrichment analysis was conducted and visualized using g:Profiler, Cytoscape, EnrichmentMap, and AutoAnnotation to determine fertility-related biological functions according to species.

**Results:**

In particular, gamete production and protein biogenesis-associated pathways were enriched in bull spermatozoa with below-normal fertility, whereas mitochondrial-associated metabolic pathways were enriched in boar spermatozoa with normal fertility. These results indicate that below-normal fertility may be determined by aberrant regulation of protein synthesis during spermatogenesis, and the modulation of reactive oxygen species generation to maintain capacitation and the acrosome reaction governs boar sperm fertility.

**Conclusion:**

Overall, this approach demonstrated that distinct molecular pathways drive sperm fertility in mammals in a species-dependent manner. Moreover, we anticipate that searching for species-specific signaling pathways may aid in the discovery of fertility-related biomarkers within large omics datasets.

**Supplementary Information:**

The online version contains supplementary material available at 10.1186/s40104-023-00836-1.

## Background

Male infertility is linked to complicated physiological and biochemical mechanisms, and basic semen analysis is insufficient for fully deciphering the underlying causes [1]. Elucidating the molecular mechanisms involved in male infertility may contribute to male fertility monitoring, diagnosis, and therapy.

Comprehensive and comparative omics studies have recently been conducted in animals and humans to provide a better understanding of the complex multifactorial processes linked to male fertility at the gene [2–5], transcript [6–8], protein [9–14], and metabolite levels [6, 15–18]. The development of new molecular methodologies to determine male fertility potential or sperm dysfunction has been facilitated by the accumulation of massive fertility-related omics datasets that serve as promising resources for identifying biomarkers of male fertility [10, 19]. However, the large scale and unbiased nature of these studies means that they inevitably include data irrelevant to fertility status; for example, most identified genes are not correlated with fertility. Hence, filtering of redundant or irrelevant data through rigorous testing and summarization of relevant data are required for field use [10]. To this end, we aimed to consolidate comparative omics data including transcriptomic, proteomic, and metabolomic from boar and bull spermatozoa with normal and below-normal fertility. We also built and visualized new pathway results as enrichment maps based on the summarized data to provide additional information on functionally associated genes, rather than focusing only on identification of valuable markers. Because bovine and porcine spermatozoa have digital fertility data following artificial insemination, which reflect a wide spectrum of field fertility level, we attempted to collect the omics data from these species for this work [10, 20].

## Materials and methods

### Data collection from transcriptomic studies

The PubMed database was searched for comprehensive and comparative research articles using the terms “transcriptomic”, “boar spermatozoa”, “bovine spermatozoa”, and “fertility”. Further relevant studies were identified by searching the reference lists of the cited articles. Differentially expressed transcripts in spermatozoa with normal or below-normal fertility were integrated according to species, and genes with inconsistent results among the studies were removed (Table 1).

**Table 1:** Criteria for the inclusion of studies in this study and summary of the reviewed publications including samples, fertility score, the applied omics approaches, detailed methods, and statistic method

Screening (Terms)	Eligibility	Included			
		Fertility	Detailed methods	Statistic	Reference
Bovine spermatozoa Fertility Transcriptomic *n* = 32	Included (*n* = 4) Excluded (*n* = 28) - Review paper (*n* = 3) - Not sperm cells - Testicular cells (*n* = 3) - Epididymis (*n* = 2) - Embryo (*n* = 3) - Not related to fertility - Motility (*n* = 3) - Cryostress (*n* = 1) - Entire sperm (*n* = 6) - No transcriptomic studies (*n* = 6) - Different species (*n* = 1)	The percentage deviation of its conception rate from the average conception of all bulls Normal > 5.1% Below-normal < − 10.8%	DNA microarray	2-fold	Feugang et al. [21]
		Field conception rates Normal > 43% Below-normal < 25%	Agilent microarray	1.5-fold	Saraf et al. [22]
		Field conception rates Normal > 40% Below-normal < 40%	Quantitative RT-PCR	Correlation	Parthipan et al. [23]
		Field conception rates Normal > 1.8 Below-normal < − 0.4	RNA Seq	2-fold	Card et al. [8]
Porcine spermatozoa Fertility Transcriptomic *n* = 21	Included (*n* = 6) Excluded (*n* = 15) - Not sperm cells - Testicular cells (*n* = 2) - Epididymis (*n* = 1) - Seminal plasma (*n* = 1) - Microbiome (*n* = 1) - Not related to fertility - Motility (*n* = 2) - Entire sperm (*n* = 1) - Quality (*n* = 2) - No transcriptomic studies (*n* = 2) Different species (*n* = 3)	The deviation of both the farrowing rate (FR) and litter size (LS) Normal: FR > 0.45, LS > 0.15 Below-normal: FR > − 0.12, LS > − 0.18	DNA microarray	Significant (*P* < 0.05)	Alvarez-Rodriguez et al. [24]
		Litter size Normal > 17 Below-normal < 17	Quantitative RT-PCR	Correlation	Pang et al. [20]
		Litter size Normal > 14.0 Below-normal < 10.8	Quantitative RT-PCR	Correlation	Kim et al. [25]
		The deviation of both the farrowing rate (FR) and litter size (LS) Normal: FR > 0.94, LS > 0.11 Below-normal: FR > − 0.42, LS > − 0.14	RNA Seq	Significant (*P* < 0.05)	Alvarez-Rodriguez et al. [24]
		The deviation of both the farrowing rate (FR) and litter size (LS) Normal: FR > 0.45, LS > 0.15 Below-normal: FR > − 0.12, LS > − 0.18	DNA microarray	Significant (*P* < 0.05)	Alvarez-Rodriguez et al. [26]
		Litter size Normal > 13.6 Below-normal < 11.2	Quantitative RT-PCR	Correlation	Kang et al. [27]
Bovine spermatozoa Fertility Proteomic *n* = 48	Included (*n* = 4) Excluded (*n* = 44) - Review paper (*n* = 6) - Not sperm cells - Seminal plasma (*n* = 10) - Testicular cells (*n* = 3) - Etc (*n* = 2) - Not related to fertility - Functionality (*n* = 4) - Cryostress (*n* = 4) - Entire sperm (*n* = 4) - Heatstress (*n* = 2) - Etc (*n* = 2) - No proteomic studies (*n* = 5) - Full text available/ Limitation to access the protein information (*n* = 2)	60-d non-return rate (NRR) Normal ≥ 70% Below-normal < 70%	2-Dimensional electrophoresis (2-DE)	2-fold	Park et al. [10]
		The percent deviation of its conception from the average conception of all bulls Normal > 5.1% Below-normal < − 10.8%	Multidimensional protein identification technology	2-fold	Peddinti et al. [28]
		Fertility solution (SOL, Zero is average fertility) Normal > 2 Below-normal < − 3.0	iTRAQ and mass spectrometry	> 1.2-fold or < 0.8-fold	D’Amours et al. [29]
		SOL Normal > 3.0 Below-normal < − 5.0	2-DE	Significant (*P* < 0.05)	D’Amours et al. [30]
Porcine spermatozoa Fertility Proteomic *n* = 35	Included (*n* = 4) Excluded (*n* = 31) - Review paper (*n* = 8) - Not sperm cells - Seminal plasma (*n* = 6) - Not related to fertility - Maturation (*n* = 3) - Functionality (*n* = 1) - Preservation (*n* = 5) - Entire spermatozoa (*n* = 2) - No proteomic studies (*n* = 4) - ETC (*n* = 2)	Litter size Normal > 12 Below-normal < 10	2-DE	3-fold	Kwon et al. [9]
		Litter size Normal > 12 Below-normal < 10	2-DE	3-fold	Kwon et al. [13]
		Litter size Normal > 11 Below-normal < 6	2-DE	4-fold	Kwon et al. [14]
		Litter size Normal > 10.8 Below-normal < 9.6	iTRAQ and strong cation-exchange chromatography (SCX) fractionation	1.2-fold	Chen et al. [31]
Bovine spermatozoa Fertility Metabolomic *n* = 9	Included (*n* = 3) Excluded (*n* = 6) - Review paper (*n* = 1) - Not sperm cells - Seminal plasma (*n* = 2) - Crossbreed (*n* = 1) - Functionality (*n* = 1) - Cryostress (*n* = 1)	Conception rate Normal > 43% Below-normal < 25%	LC-MS/MS analysis	Significant (*P* < 0.05)	Saraf et al. [17]
		In vitro fertilization rate	LC-MS/MS analysis	2-fold	Longobardi et al. [18]
		The percent deviation of its conception from the average conception of all bulls Normal > 3.6% Below-normal < − 3.8%	GC-MS	Significant (*P* < 0.05)	Menezes et al. [6]

### Data collection from proteomic studies

The PubMed database was searched for comprehensive and comparative research articles using the terms “proteomic”, “porcine spermatozoa”, “bovine spermatozoa”, and “fertility”. According to species, differentially expressed proteins in spermatozoa with normal or below-normal fertility were integrated, and genes having contradictory results across studies were eliminated.

To evaluate the fertility-related proteomic enrichment pathways, four comparative and comprehensive proteomic studies in spermatozoa between normal (high or fertile) and below-normal (low or infertile) fertility bulls were considered for evaluation [10, 28–30]. In these studies, bull spermatozoa represented a wide range of field fertility levels (60% ~ 90% of non-return rate) and at least 16% [28] to 34% of differences between normal and below-normal fertility were detected [10].

### Data collection from metabolomic studies

The PubMed database was searched for comprehensive and comparative research articles using the terms “metabolomic”, “porcine spermatozoa”, “bovine spermatozoa”, and “fertility”. Further relevant studies were identified by searching the reference lists of the cited articles. Differentially expressed metabolites in spermatozoa with normal or below-normal fertility were integrated according to species, and genes with inconsistent results among the studies were removed.

### Pathway enrichment analysis and visualization

Pathway enrichment analysis was performed by following a step-by-step procedure to facilitate the interpretation of transcriptome and proteome data as described by Reimand et al. [32] with some modifications. All transcript and protein lists of interest were investigated using the pathway enrichment analysis with g:Profiler, which searches a collection of gene sets representing Gene Ontology (GO) terms, pathways, and networks. The AutoAnnotate Cytoscape tool was used to automatically annotate pathways based on the GO, Kyoto Encyclopedia of Genes and Genomes (KEGG), and Reactome databases to obtain similar groupings representing significant biological themes (Version 3.8.2).

A list of differentially expressed metabolites between normal and below-normal fertility bull spermatozoa was compiled, and enriched pathways were examined using Metabolite Set Enrichment Analysis from MetaboAnalyst 4.0 (www.metaboanalyst.ca).

### Sample preparation

Frozen semen samples from ten bulls (Hanwoo, Korean native cattle) that represented a wide range of filed fertility levels were obtained from the Hanwoo Improvement Program of the National Agriculture Cooperative Federation (NACF) of Korea. According to our previous study [10], the 60 d non-return rate (NRR) was used as the indicator of bull fertility, and the ten bulls were divided into two groups; a normal fertility group (80.49 ± 2.92, *n* = 5) and a below-normal fertility (57.00 ± 2.73, *n* = 5). NRR was calculated from recent 3-year of historical data had at least 100 breeding with at least ten herds.

Liquid semen samples from six boars (Yorkshire) with known litter size were obtained from Grand-Grand Parents farm (Sunjin Co., Danyang Korea). Based on the average litter size, ten boar semen samples were divided into normal (15.26 ± 0.08, *n* = 3) and below-normal fertility (13.34 ± 0.23, *n* = 3). Average litter size was calculated based on the records of at least 100 sows by artificial insemination.

### Western blot analysis

Western blot analysis was conducted with the pooled semen samples from normal and below-normal fertility spermatozoa to minimize individual differences. Moreover, the analysis was repeated at least three times with sperm samples from another set of semen batches from each group. Based on the signaling pathway enrichment analysis, we tried to determine whether the species-specific or fertility-related proteins are differentially expressed between normal and below-normal fertility using Western blot analysis. Both bovine and porcine semen samples were subjected to discontinuous Percoll separation in accordance with our previous studies [10, 13]. The sperm cells (1 × 10^6^ cells) were lysates with Laemmli sample buffer consisting 63 mmol/L Tris-Cl, 10% glycerol, 10% sodium dodecyl sulphate (SDS), 5% bromophenol blue, and 5% mercaptoethanol. Total sperm cell lysates were loaded into 12% SDS-polyacrylamide gel and transferred to polyvinylidene difluoride membranes (Amersham Bioscience Corp., Amersham, UK). And then membranes were blocked with 5% skim milk, and incubated with primary antibodies against enolase 1 (ENO1), glucose transporter 3 (GLUT3), glutathione peroxidase 4 (GPX4), cytochrome b-c1 complex subunit 1, mitochondrial (UQCRC1), UQCRC2, NADH:Ubiquinone oxidoreductase core subunit S8 (NDUFS8), ATP synthase, H^+^ transporting, mitochondrial F0 complex (ATP5F), voltage-dependent anion channel 2 (VDAC2), actin-related protein T2 (ACTRT2), cytochrome c oxidase polypeptide 5, mitochondrial (COX5) A, COX5B, COX6B, parkinsonism-associated deglycase (PARK7), cytochrome C (CYC1), and α-tubulin. All antibodies were purchased from Abcam (Abcam, Cambridge, MA, USA). Protein expression was visualized with Clarity Western ECL substrate (Bio-Rad, CA, USA) and quantified using ImageJ software (National Institutes of Health, Bethesda, MD, USA).

## Results

### Functional annotation and enrichment maps of fertility-related transcriptomes

We summarized the significant differentially expressed fertility-related transcriptomic markers to construct comprehensive and novel functional enrichment maps. Based on the previous transcriptomic studies, a total 470 transcripts were identified as a subset of transcripts that are possibly relevant for bovine sperm fertility [8, 21–23, 33]. One hundred and forty-three transcripts were highly expressed in spermatozoa from normal fertility bulls, while 327 transcripts were abundant in spermatozoa from below-normal fertility bulls (Table 1 and Additional file 1: Table S1). A total 74 transcripts including 68 of abundant in normal-fertility and 5 transcripts of highly expressed in below-normal fertility porcine spermatozoa was identified from 6 transcriptomic studies [24–27, 34] and used to gain a deep functional fertility-related insights (Table 1 and Additional file 1: Table S1).

**Fig. 1:**
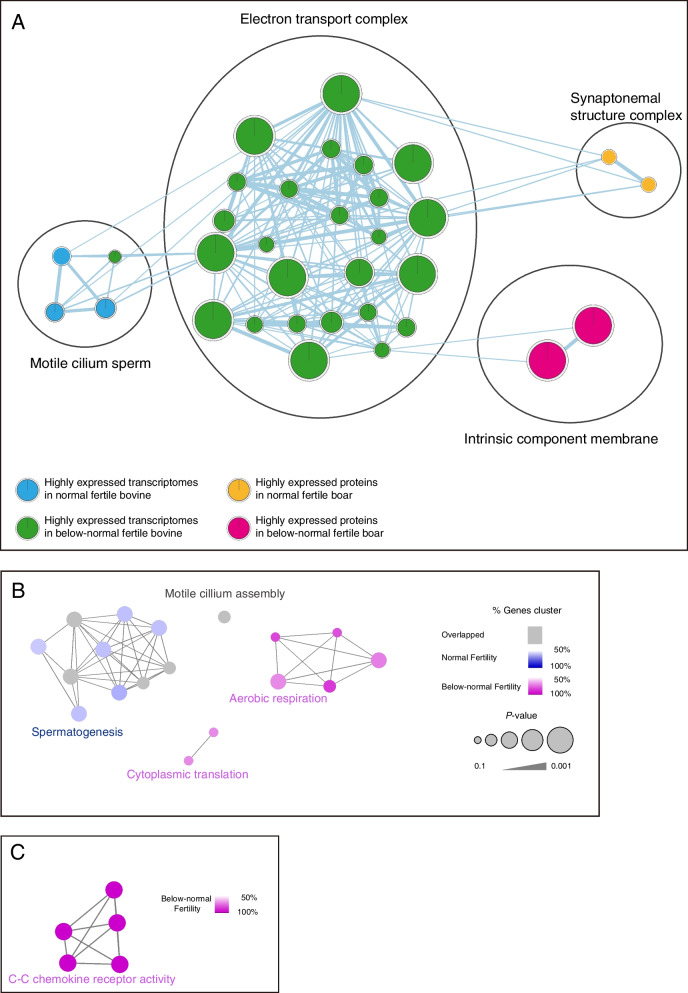
Signaling pathways from transcripts differentially expressed between bull and boar spermatozoa with normal and below-normal fertility. **A** Enrichment map created based on the differentially expressed transcripts in bull and boar spermatozoa with normal and below-normal fertility. Blue and yellow, enrichment in bovine and porcine spermatozoa with normal fertility, respectively; green and pink, enrichment in bovine spermatozoa with below-normal fertility. Biological Process GO terms based on the differentially expressed transcripts in (**B**) bovine and (**C**) porcine spermatozoa with normal and below-normal fertility. Blue, enrichment in spermatozoa with normal fertility; pink, enrichment in spermatozoa with below-normal fertility. Enrichment maps were created using g:Profiler, Cytoscape, EnrichmentMap, and ClueGO with FDR Q < 0.05

Pathway enrichment analyses of markers that were significantly differentially expressed between spermatozoa with normal and below-normal fertility from bulls (Additional file 1: Table S1) or boars (Additional file 1: Table S2) were conducted using g:Profiler and visualized by EnrichmentMap and ClueGO application of Cytoscape. Pathways with similar biological functions were automatically defined and clustered on the enrichment maps using the AutoAnnotate Cytoscape application, and the significance of the enrichment of each pathway was determined using the false discovery rate (FDR; Fig. 1). Gene Ontology terms was visualized with EnrichmentMap of Cytoscape to identify the species-specific Biological Process according to their fertility. In bovine, the ‘motile cilium sperm’ was clustered together in the bovine spermatozoa from normal fertility group, while one enriched pathway, ‘electron transport complex’, was clustered only in the spermatozoa from below-normal fertility group (Fig. 1A, FDR < 0.05). Moreover, ClueGO analysis revealed that the ‘spermatogenesis’ signaling pathway was uniquely upregulated in bovine spermatozoa from normal fertility group, while two Biological Process GO terms, including aerobic respiration and cytoplasmic translation was upregulated in bovine spermatozoa from below-normal fertility group (Fig. 1B). Otherwise, the ‘synaptonemal structure complex’ associated Biological Process GO term was upregulated in boar spermatozoa from normal fertility group, while the ‘intrinsic component membrane’ related enrichment pathway was significantly enriched in the porcine spermatozoa from below-normal fertility group (Fig. 1A, FDR < 0.05). In ClueGO analysis of porcine spermatozoa, we did not detect any upregulated Biological Process GO terms in normal fertility group. However, only one enrichment pathway, C-C chemokine receptor activity, was identified in the below-normal fertility porcine spermatozoa (Fig. 1C, FDR < 0.05).

### Functional annotation and enrichment maps of fertility-related proteomes

Following the filter out of hypothetical or predicted proteins, a total of 56 proteins, including 33 proteins highly expressed in normal fertility bull spermatozoa and 23 proteins highly expressed in below-normal fertility bull spermatozoa, were taken into consideration for the pathway enrichment analysis. Four comparative proteomic studies were considered for evaluation of fertility-related signaling pathways in boar spermatozoa [9, 13, 31, 35] (Table 1 and Additional file 1: Table S3). Litter size was used as an indicator of boar sperm fertility and the litter size for the normal and below-normal fertility boar spermatozoa were more than 10.8 and less than 10.19, respectively (Table 1 and Additional file 1: Table S4). For pathway enrichment analysis, 147 proteins were accounted, including 98 proteins that are abundant in boar spermatozoa with normal fertility and 49 proteins that are abundant in spermatozoa with below-normal fertility.

To analyze the GO enrichment pathways associated with fertility-related proteins, differentially expressed proteins between spermatozoa with normal and below-normal fertility from bulls (Additional file 1: Table S3) or boars (Additional file 1: Table S4) were used to explore the major Biological Process GO enrichment pathways using g:Profiler. The pathways were visualized and annotated using Enrichment Map and ClueGO based on the GO, KEGG, and Reactome databases.

**Fig. 2:**
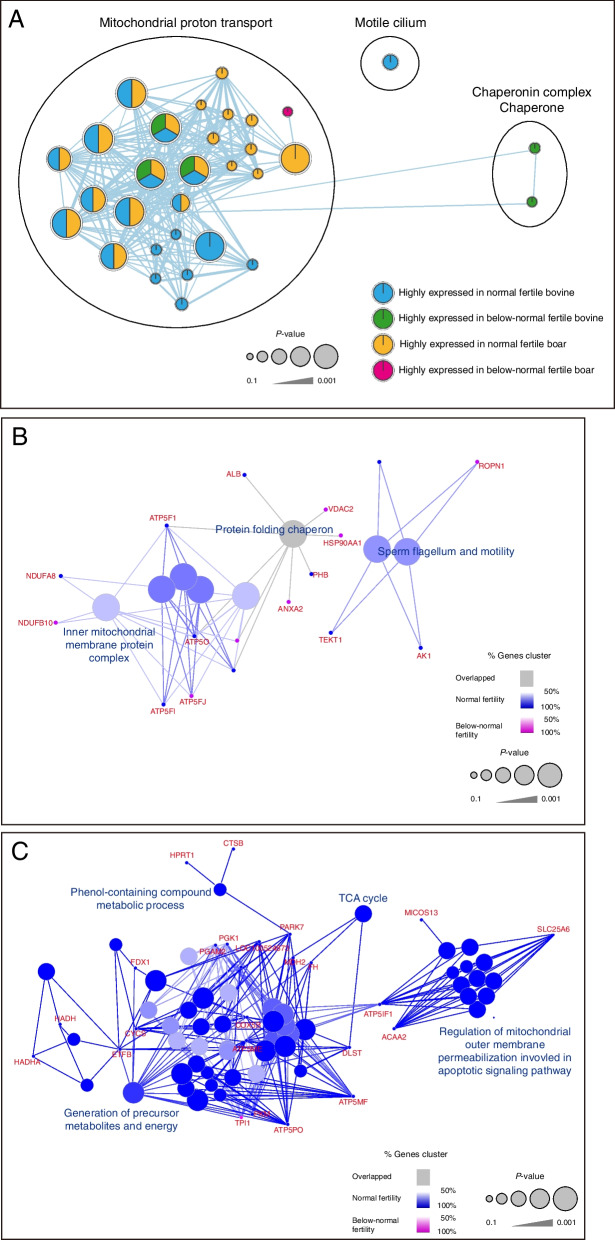
Important signaling pathway-related to proteins differentially expressed between bull or boar spermatozoa with normal and below-normal fertility. **A** Integration of an enrichment map of differentially expressed proteins in spermatozoa from bulls and boars with normal and below-normal fertility. Blue, enrichment in bull spermatozoa with normal fertility; green, enrichment in bull spermatozoa with below-normal fertility; yellow, enrichment in boar spermatozoa with normal fertility; pink, enrichment in boar spermatozoa with below-normal fertility. **B** Biological Process GO terms based on the differentially expressed proteins in (**B**) bovine and (**C**) porcine spermatozoa with normal and below-normal fertility. Blue, enrichment in spermatozoa with normal fertility; pink, enrichment in spermatozoa with below-normal fertility. The enrichment map was created using g:Profiler, Cytoscape, and EnrichmentMap with FDR Q < 0.01

As shown in Fig. 2A, the ‘motile cilium’ associated enriched pathway was upregulated in normal fertility bovine spermatozoa, while ‘chaperonin complex chaperone’ was upregulated in below-normal fertility group. The ‘mitochondrial proton transport’ enrichment pathway was clustered together in bovine and porcine spermatozoa regardless fertility. However, we did not detect any specific enrichment pathway in boar spermatozoa (Fig. 2A, FDR < 0.05). Among the varied ‘mitochondrial proton transport’ related enrichment pathways, four enriched clusters, including ‘acid oxoacid metabolic’, ‘carbohydrate derivative’, ‘electron transport chain’, and ‘lactate metabolic process’ were upregulated in porcine spermatozoa from normal fertility group (Fig. 2B, FDR < 0.05). Otherwise, only one enrichment pathway ‘phospholipase A2 activity’ was clustered in porcine spermatozoa from below-normal fertility group (Fig. 2B, FDR < 0.05).

### Differential fertility-related protein level in bovine and porcine spermatozoa

**Fig. 3:**
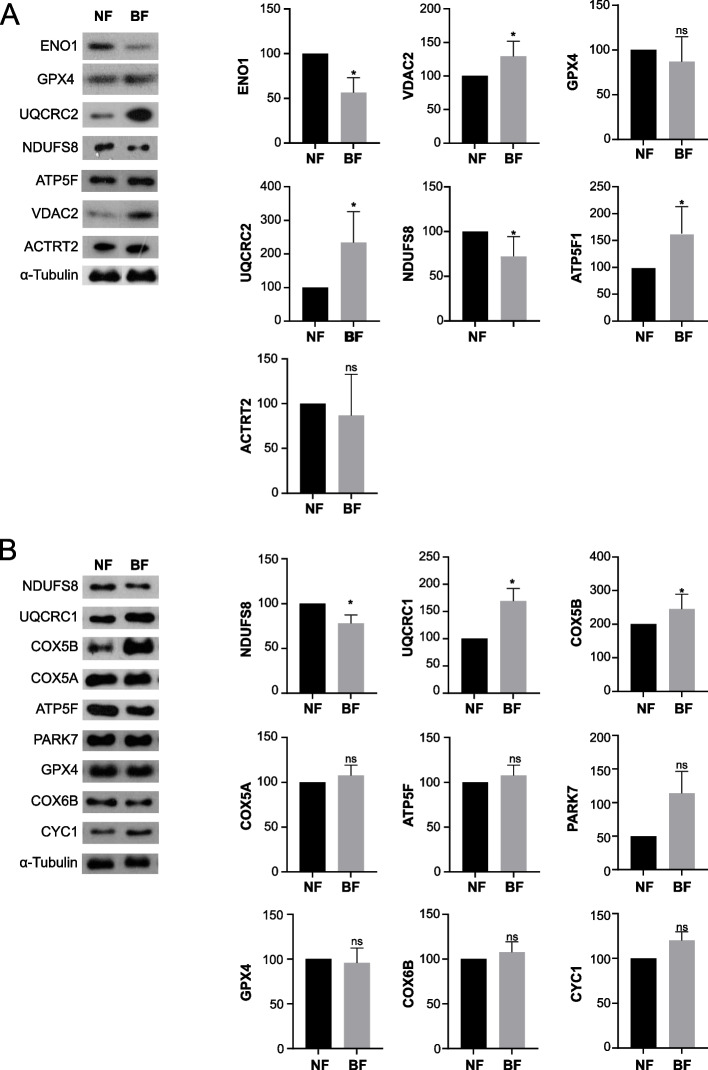
Enrichment signaling pathway related protein expression in the spermatozoa. Different expression patterns of proteins annotated with enrichment signaling pathways in (**A**) bovine and (**B**) porcine spermatozoa between normal and below-normal fertility group were analyzed by western blot. Data represent the mean of three experiments ± standard error of the mean (SEM)

As shown in Fig. 2, the ‘mitochondrial proton transport’ was annotated together in the bovine and porcine spermatozoa regardless their fertility. Therefore, we tried to analyze the different mitochondrial protein level in spermatozoa between normal and below-normal fertility group. The ‘inner mitochondrial membrane protein complex’ was highly represented in proteins, including NDUFs, ATP synthase subunits, and UQCRC2, differentially expressed in bovine spermatozoa according to fertility (Fig. 2B, FDR < 0.05). Similarly, the NDUFS8 protein level was upregulated in bovine spermatozoa from normal fertility group, while UQCRC2 and ATP5F protein level were upregulated in spermatozoa from below-normal fertility group (Fig. 3A, *P* < 0.05). Moreover, sperm flagellum associated with enrichment pathways, including sperm flagellum and myelin sheath, were most highly enriched in fertility-related proteins in bovine spermatozoa (Fig. 2B, FDR < 0.05). VDAC2, which is a sperm flagellum and myelin sheath marker, was upregulated in bovine spermatozoa from below-normal fertility group (Fig. 3A, *P* < 0.05). Also, VDAC2, ENO1, and GPX4 proteins were used as the spermatogenesis markers [36–38] which was uniquely clustered in fertility related transcriptomes from bovine spermatozoa (Fig. 1B). Although ENO1 protein level was upregulated in bovine spermatozoa from normal fertility group, there was no significant difference in GPX4 level of bovine spermatozoa between normal and below-normal group (Fig. 3A).

In porcine spermatozoa from normal fertility group, four Biological Process GO terms, including ‘phenol-containing compound metabolic process’, ‘TCA cycle’, ‘generation of precursor metabolites and energy’, and ‘regulation of mitochondrial outer membrane permeabilization involved in apoptotic signaling pathway’ were identified by ClueGO analysis (Fig. 2C). However, no enrichment pathway was detected in below-normal fertility group. Based on the enrichment pathways, the level of nine mitochondrial related proteins, including NDUFS8, UQCRC1, COX5B, COX5A, ATP5F, PARK7, GPX4, COX6B, and CYC1, were compared between normal and below-normal fertility group. Only three proteins showed that different level according to fertility; one protein, NDUFS8, was upregulated in normal fertility group, while two proteins, including UQCRC1 and COX5B, were upregulated in below-normal fertility group (Fig. 3B, *P* < 0.05).

### Functional annotation and enrichment maps of fertility-related metabolomes

A total of 32 metabolites including 17 metabolomes in highly expressed in normal fertility and 15 metabolomes in highly expressed in below-normal fertility was identified from 3 comparative metabolomic studies [6, 17, 18] and used for better understanding of fertility-related functional insights (Table 1 and Additional file 1: Table S5).

We compiled a list of the metabolomes that were differentially expressed between bull spermatozoa with normal and below-normal fertility (Additional file 1: Table S5) and used MetaboAnalyst 4.0 to examine the enriched pathways (Fig. 3). Pyruvate metabolism and glycolysis-associated metabolites were highly upregulated in bull spermatozoa with normal fertility, whereas fatty acid metabolism and oxidation-related metabolites were upregulated in bull spermatozoa with below-normal fertility (Fig. 3).

## Discussion

**Fig. 4:**
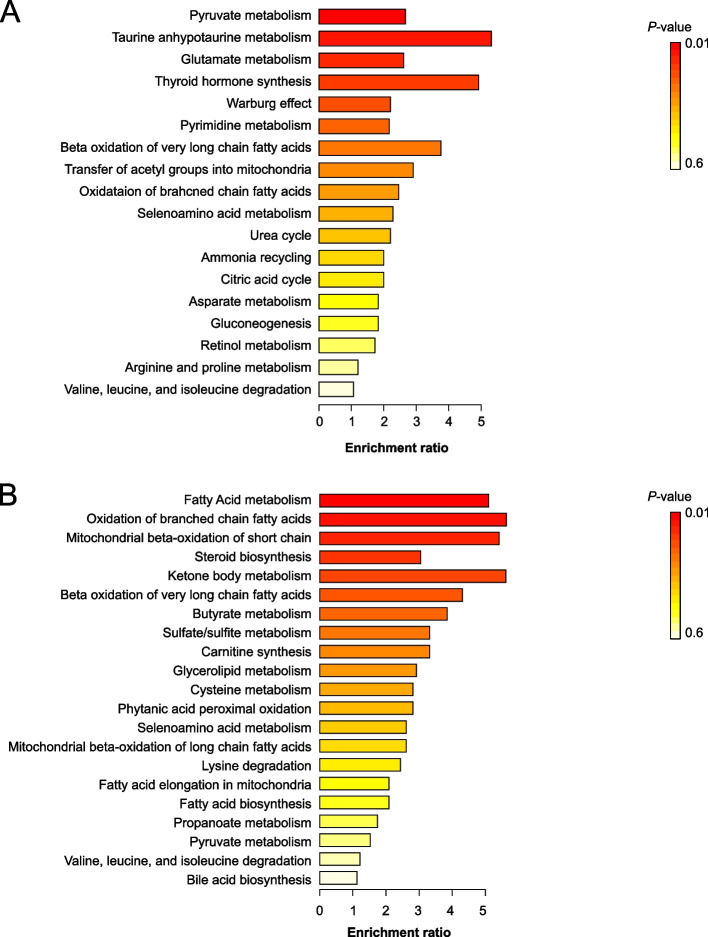
Metabolite set enrichment analysis of metabolites differentially expressed between bull spermatozoa with normal and below-normal fertility. Functional enrichment analysis of pathways from bull spermatozoa with normal **(A)** and below-normal **(B)** fertility. MetaboAnalyst 4.0 was used to examine and interpret the enrichment pathways. The color depth and column length indicate the degree of disturbance

In depth omics studies have recently been carried out in human and animal spermatozoa to provide a better understanding of male fertility at the genes [2–5], transcript [6–8], protein [9–14], and metabolite levels [6, 11, 15–18]. Although these huge fertility-related omics datasets can offer prospective sources for discovering male fertility biomarkers which contribute to assess male fertility potential or sperm dysfunctions, these studies invariably contain information that is unrelated to male fertility status. Therefore, it is necessary to filter out redundant or irrelevant data through rigorous testing and to summarize pertinent data [39]. Therefore, this study was to accumulate the fertility-related transcriptomic, proteomic, and metabolomic data from boar and bull spermatozoa. Moreover, rather than focusing only on the identification of valuable markers associated with male fertility, enrichment maps based on the consolidated omics data were analyzed to provide additional information on functionally linked genes and contribute to a comprehensive understanding of fertility-related mechanisms at the gene, transcript, protein, and metabolite levels (Fig. 4).

Although spermatozoa are transcriptionally and translationally dormant cells after leaving the testis, residual RNA produced in the early stages of spermatogenesis stays in spermatozoa and can be transmitted to oocytes to deliver the paternal epigenetic information [40, 41]. Various types of RNAs, such as messenger RNA, ribosomal RNA, mitochondrial RNA, long non-coding RNA, small non-coding RNA, and transfer RNA, contribute to sperm morphological and functional parameters, embryonic development, and pregnancy outcomes, as determined using recently developed transcriptomic analyses [7, 24, 42, 43]. Moreover, comparative whole-transcriptome sequencing analysis of spermatozoa with normal and below-normal fertility has provided novel molecular markers for evaluating the fertilizing potential of spermatozoa [7, 8, 21, 23, 24, 33]. Indeed, their lack of transcription and translation coupled with their abundant, highly specialized, compartmentalized nature makes mature spermatozoa a useful model for proteomic analysis of fertility [1, 44]. On the basis of these studies, we investigated the significant fertility-related transcriptomic and proteomic markers to establish comprehensive and novel functional enrichment maps. Our integrated transcriptomic and proteomic data showed that mitochondrial-associated biological processes are closely related to sperm fertility, regardless of species. Among the various mitochondrial-associated signaling pathways, the inner mitochondrial membrane protein complex related to proton transport-associated enrichment pathways was positively related to bovine fertility. Moreover, the TCA cycle, generation of energy metabolites, and mitochondrial function related to apoptotic signaling pathways were positively associated with boar sperm fertility. Respiratory pathways, including glycolysis, the TCA cycle, and mitochondrial electron transport chain, are essential for energy provision in spermatozoa to maintain their viability, motility, and fertilizing ability [39]. Although glycolysis in the principal piece of spermatozoa is one of the main sources of ATP for supporting motility, the massive ATP production required for regulating sperm motility, capacitation, and chromatin integrity is produced via OXPHOS in the mitochondria [45]. Activation of OXPHOS through sequential electron transfer in the inner mitochondrial membrane facilitates ATP production, and ROS are necessary for capacitation and fertilization [46]. TCA- and OXPHOS-connected signaling pathways were enriched in both bull and boar spermatozoa with normal fertility, corroborating with the results of our previous study, which showed that TCA cycle signaling pathway-related proteins were substantially expressed in spermatozoa from fertile males compared to those from infertile males [39]. However, excessive ROS production can cause lipid peroxidation and DNA damage in spermatozoa, leading to infertility. ROS also reduce the intracellular ATP content, which leads to a decrease in flagellar beat frequency, resulting in sperm motility loss [46, 47]., Additionally, spermatozoa with downregulated respiratory pathways may exhibit a reduced capacity for preventing damage caused by ROS, resulting in a decrease in fertility. Therefore, we hypothesize that the regulation of ATP and ROS levels through the well-orchestrated respiratory pathways in spermatozoa may be important for maintaining sperm fertility in mammalian spermatozoa.

Moreover, our integrated omics studies represented that sperm flagellum and motility associated enrichment pathways were positively related to bovine fertility. Following glycolysis and the TCA cycle, OXPHOS occurs in the mitochondrial-rich midpiece of spermatozoa, generating large amounts of ATP and reactive oxygen species (ROS). ATP and ROS are both required for boosting the hyperactivation of spermatozoa during capture and fertilization; ROS stimulate adenylyl cyclase to catalyze the synthesis of cyclic AMP from ATP, leading to protein kinase A activation, which stimulates the phosphorylation of tyrosine residues in the fibrous sheath of the sperm flagellum, resulting in hyperactivation of spermatozoa [48, 49]. Moreover, ROS regulate calcium efflux and membrane fluidity to facilitate the sperm–oocyte fusion and fertilization [50–52]. Therefore, we postulate that fine-tuning ROS and ATP synthesis during sperm capacitation and fertilization may control the fertility of bull spermatozoa.

It is noteworthy that spermatogenesis and structure-associated signaling pathways, such as chaperonin complex associated- and protein folding-related signaling pathways, were specifically identified in bovine spermatozoa, and negatively related to bovine fertility. The endoplasmic reticulum is an essential cellular component that plays a vital role in the folding and assembly of newly synthesized proteins in mammalian cells. However, because the endoplasmic reticulum is eliminated during spermatogenesis, a highly active protein synthesis and folding event occurs before the completion of spermatogenesis [53]. Thus, we suggest that the upregulation of protein folding pathways in bull spermatozoa with below-normal fertility may reflect the aberrant protein folding during spermatogenesis, which ultimately determines low fertility in bull spermatozoa. This hypothesis is in line with our previous research, which showed that male fertility factors, such as sperm motility, are determined in the testes or epididymis before spermatozoa have fully matured [54].

The female reproductive tract contains many chemokines that trigger chemotaxis to control sperm motility and capacitation to optimize contact with oocytes [55, 56]. Several chemokine receptors, including CCR5 and CCR6 were identified in the spermatozoa, which induce chemoattraction before fertilization [57, 58]. Especially, sperm chemotaxis is regulated by calcium efflux through the plasma membrane calcium ATPase, which works as the sperm-activating and attracting factor [59]. An increase chemotaxis in spermatozoa through the increase of the intracellular calcium level accelerated the hyperactivation, capacitation, and the acrosome reaction [59, 60]. Although the role of chemotaxis reaction in spermatozoa is proposed as the essential process for capacitation and fertilization, the chemokine receptor activity-related enrichment pathway is negatively related to sperm fertility. Collectively, these findings led us to hypothesize that upregulation of chemokine receptor activity in boar spermatozoa might elevate the chemotaxis behavior, resulting in premature capacitation and infertility.

The dynamic relationships between genes, transcripts, proteins, and metabolites allow biological systems to function as a cohesive unit. Metabolites play an essential role in the biochemical environment because they serve as the primary components of all other biochemical structures, such as proteins, genes, and transcripts [61]. Metabolomics is a key scientific field in the post-genomics era that investigates small molecules to complement genomic, proteomic, and transcriptomics and aids in the identification of novel disease biomarkers and therapeutic strategies [62]. Metabolomic analysis has also been used in the field of male infertility research to identify fertility-related metabolomic markers in spermatozoa or seminal plasma [63, 64]. Although most metabolomic investigations have been conducted in humans, we were able to find three comprehensive and comparative metabolomic studies on bull spermatozoa [6, 17, 18]. By reanalyzing these datasets, we found that pyruvate metabolism and glycolysis-associated metabolites were highly upregulated in bull spermatozoa with normal fertility, whereas fatty acid metabolism and oxidation-related metabolites were upregulated in bull spermatozoa with below-normal fertility. During spermatogenesis, energy production systems shift from glycolysis to OXPHOS depending on the ATP requirement of the cell [65]. Although OXPHOS is a more efficient pathway for ATP production than glycolysis, glycolysis is also required for maintaining sperm motility in the principal piece. Thus, we anticipated that spermatozoa with downregulated glycolysis would be unable to generate sufficient ATP to maintain sperm motility, resulting in below-normal fertility. Following epididymal maturation, the proportion of polyunsaturated fatty acids (PUFAs) in the sperm plasma membrane is increased to enhance membrane integrity and fusion ability for fertilization; however, this also enhances the sensitivity of spermatozoa to oxidative stress [66, 67]. When ROS production exceeds the capacity of the antioxidant defense system, PUFA-containing spermatozoa undergo lipid peroxidation. Thus, PUFAs protect cells from damage caused by high levels of ROS, which cause changes in membrane structure, function, and permeability [68]. However, because moderate ROS levels are required for sperm capacitation, the acrosome reaction, and fertilization, we hypothesize that upregulation of fatty acid levels in the sperm membrane may impair these processes, resulting in a loss of fertility.

## Conclusions

This study was to accumulate fertility-related omics data from boar and bull spermatozoa to provide comprehensive fertility-related mechanisms from gene to protein levels. Mitochondrial-associated signaling pathways were commonly identified as the significant fertility-related signaling pathways in both species; we also found species-specific pathways. Our analyses indicate the proper synthesis and folding of proteins during spermatogenesis may determine the bull sperm fertility. In contrast, the modulation of chemotaxis activity may determine boar sperm fertility to maintain the optimal capacitation state and the acrosome reaction. Although the further study is required for confirmation of different gene expression by western blot and RT-PCR between normal and below-normal spermatozoa to identify specific species-dependent mechanisms, this study provide preliminary information about different molecular pathways that govern sperm fertility in mammals depending on the species. Therefore, we propose that screening for species-specific signaling pathways may help identify fertility-related biomarkers within massive omics data. Also, this study may be useful for addressing species-specific fertility control systems to improve animal productivity. Furthermore, employing such evaluation processes may contribute to the elucidation of yet unknown characteristics of spermatozoa, which may contribute to enhancing reproduction.

## Supplementary Information

**Additional file 1: Table S1.** Summary of differentially expressed transcripts in bull spermatozoa with normal and below-normal fertility. **Table S2.** Summary of differentially expressed transcripts in boar spermatozoa with normal and below-normal fertility. **Table S3.** Summary of differentially expressed proteins in bull spermatozoa with normal and below-normal fertility. **Table S4.** Summary of differentially expressed proteins in boar spermatozoa with normal and below-normal fertility. **Table S5.** Summary of differentially expressed metabolites in bovine spermatozoa with normal and below-normal fertility.

## Data Availability

Not applicable.

## Abbreviations

ACTRT2: Actin-related protein T2
ATP5F: ATP synthase, H^+^ transporting, mitochondrial F0 complex
COX5: Cytochrome c oxidase polypeptide 5, mitochondrial
CYC1: Cytochrome C
ENO1: Enolase 1
FDR: False discovery rate
GLUT3: Glucose transporter 3
GO: Gene Ontology
GPX4: Glutathione peroxidase 4
KEGG: Kyoto Encyclopedia of Genes and Genomes
mGlu receptor: Metabotropic glutamate receptor
NDUFS8: NADH:Ubiquinone oxidoreductase core subunit S8
OXPHOS: Oxidative phosphorylation
PARK7: Parkinsonism-associated deglycase
PUFAs: Polyunsaturated fatty acids
ROS: Reactive oxygen species
TCA: Tricarboxylic acid
UQCRC1: Cytochrome b-c1 complex subunit 1
VDAC2: Voltage-dependent anion channel 2
