# Supplementary material for: Integration of omics studies indicates that species-dependent molecular mechanisms govern male fertility
Source: J Anim Sci Biotechnol. 2023 Mar 2;14:28. doi: 10.1186/s40104-023-00836-1 (PMC9979430; doi:10.1186/s40104-023-00836-1)
Supplement: Supplementary file 1 — Additional file 1: Table S1. Summary of differentially expressed transcripts in bull spermatozoa with normal and below-normal fertility. Table S2. Summary of differentially expressed transcripts in boar spermatozoa with normal and below-normal fertility. Table S3. Summary of differentially expressed proteins in bull spermatozoa with normal and below-normal fertility. Table S4. Summary of differentially expressed proteins in boar spermatozoa with normal and below-normal fertility. Table S5. Summary of differentially expressed metabolites in bovine spermatozoa with normal and below-normal fertility. [file 40104_2023_836_MOESM1_ESM.docx]

**Additional File 1**

**Table S1** Summary of differentially expressed transcripts in bull spermatozoa with normal and below-normal fertility

| **Highly expressed in bull spermatozoa with normal fertility** | **Highly expressed in bull spermatozoa with below-normal fertility** |
| --- | --- |
| 1-acylglycerol-3-phosphate O-acyltransferase 9  39S ribosomal protein L20, mitochondrial  40S ribosomal protein S25  60S ribosomal protein l36a  Acidic (leucine-rich) nuclear phosphoprotein 32 family, B  Actin-like 7A  Adenosylhomocysteinase  AF4/FMR2 family, member 4  Ankyrin repeat and SOCS box containing 7  Ankyrin repeat and SOCS box-containing 12  Ankyrin repeat and SOCS box-containing 8  Ankyrin repeat domain 32  Apolipoprotein O  ATP synthase membrane subunit DAPIT, mitochondrial  ATP synthase, H+ transporting, mitochondrial F0 complex  B-cell translocation gene 1, anti-proliferative  BCL2 like 11  Biphenyl hydrolase-like  Branched chain keto acid dehydrogenase E1, beta  Bromodomain adjacent to zinc finger domain, 1A  BTB  Calcium channel, voltage-dependent, beta 2 subunit  Calcium-binding and spermatid-specific protein 1  Casein kappa  CCHC-type zinc finger, nucleic acid binding protein  CD52 molecule  Cell division cycle 27 homolog  Centrin, EF-hand protein, 2  Centrosomal protein 76 kDa  Centrosome and spindle pole associated protein 1  Chromatin accessibility complex 1  Chromatin modifying protein 5  Chromosome 9 open reading frame 16 ortholog  Chromosome 9 open reading frame 9 ortholog  Citrate synthase  Claudin 1  Cofilin 1  Coiled-coil domain containing 135  Coiled-coil domain containing 67  Coiled-coil domain containing 70  Coiled-coil domain-containing protein 54  COP9 constitutive photomorphogenic homolog subunit 3  Cyclin-dependent kinase-like 4  Cytochrome b5 reductase 1  DCN1-like protein  Dnaj  Dynein, light chain, roadblock-type 2  Enhancer of yellow 2 homolog  Erythrocyte membrane protein band 4.9  Eukaryotic translation initiation factor 3, subunit G  Eukaryotic translation initiation factor 5A  Exosome complex component RRP4  F-actin-capping protein subunit alpha  Family with sequence similarity 76, member A  F-box and leucine-rich repeat protein 2  F-box and leucine-rich repeat protein 6  Fibrinogen alpha chain  Frizzled homolog 7  Fructosamine 3 kinase related protein  Gametocyte specific factor 1  Gamma-aminobutyric acid receptor-associated protein  Glutamate-rich protein 2  Glutathione S-transferase omega 2  Glycerophosphodiester phosphodiesterase 1  Glyoxalase domain containing 4  Golgi SNAP receptor complex member 1  Heat shock 70 kDa protein 3  Hemogen  High mobility group protein B4  Hippocampus abundant transcript-like protein 1  Interleukin enhancer binding factor 2, 45 kDa  IQ motif containing F1  IQ motif containing F5  J domain-containing protein  Kelch-like 24  Limbic system-associated membrane protein  Lipoic acid synthetase  LSM12 homolog  Lu-ECAM-1  Maelstrom homolog  MARCKS-like 1  Methylmalonic aciduria  Mitochondrial ribosomal protein L42  Mitogen-activated protein kinase kinase kinase kinase 3  Myeloid leukemia factor 1  Neurensin 1  Non-protein coding RNA 153  Nuclear receptor coactivator 5GPSM2  Pinin, desmosome associated protein  Pleckstrin homology domain containing, family Q member 1  Polyubiquitin-B [Cleaved into: Ubiquitin]  Proline rich 13  Prostaglandin reductase 1  Proteasome  Protein BEX3  RAB interacting factor  Receptor accessory protein 6  Ribosomal biogenesis factor  Ribosomal protein L23  Ribosomal protein S27-like  RNA binding motif protein 4B  RNA component of mitochondrial RNA processing  Ropporin 1-like  SCP2 sterol-binding domain-containing protein 1  Serine/threonine-protein kinase 38  Similar to cyclin B2; cyclin B2  Small EDRK-rich factor 2  Small nuclear ribonucleoprotein D2 polypeptide 16.5 kDa  SMT3 suppressor of mif two 3 homolog 2  Solute carrier family 2  Sperm associated antigen 17  Spermatid nuclear transition protein 1  Spermatogenesis associated 6  Spermatogenic leucine zipper 1  Succinate dehydrogenase complex, subunit B, iron sulfur  SUMO1/sentrin specific peptidase 6  Synaptophysin-like 1  T-cell immunoglobulin and mucin domain containing 4  Tektin 1  Testis-specific serine kinase 3  Thioredoxin-like 1  Transducin beta like 1 X-linked  Transformer 2 beta homolog  Transmembrane protein 30A  Ubiquitin specific peptidase 16  Ubiquitin-conjugating enzyme E2H  Ubiquitin-conjugating enzyme E2N  UBX domain protein 6  UDP-N-acetyl-alpha-d-galactosamine  UDP-N-acetyl-alpha-d-galactosamine  Vimentin  Voltage-dependent anion channel 2  WBP2 N-terminal like  WD repeat domain 74  Yippee-like 2  Zinc finger protein 24  Zinc finger protein 33B  Zinc finger protein 474  Zinc finger, CCHC domain containing 6 | 1-acylglycerol-3-phosphate O-acyltransferase 3  26S proteasome complex subunit SEM1  60S ribosomal protein L36  A kinase (PRKA) anchor protein 4  Actin related protein M1  Acyl-COA Dehydrogenase, Very Long Chain  Acyl-coa thioesterase 7  ADAM metallopeptidase domain 32  ADAM metallopeptidase domain 3A  Adaptor-related protein complex 2, beta 1 subunit  Adenosine A3 receptor  Adenosylmethionine decarboxylase 1  ADP ribosylation factor 4  ADP-ribosylation factor-like 2 binding protein  ADP-ribosylation factor-like 3  ADP-ribosylation factor-like 4A  AKT interacting protein  Allograft inflammatory factor 1  Ankyrin repeat and SOCS box-containing 17  Archaelysin family metallopeptidase 2  Arginine and glutamate rich 1  Arrestin domain containing 4  ATP synthase F1 subunit  ATP synthase membrane subunit  ATP synthase, H+ transporting, mitochondrial  Barrier to autointegration factor 2  Basic leucine zipper and W2 domains 1  Basic leucine zipper nuclear factor 1  B-cell receptor-associated protein 29  BCL2-associated athanogene 4  Bcl2-like 14  BPI fold containing family A member 3  Bromodomain adjacent to zinc finger domain, 2B  Bromodomain containing 2  C1D nuclear receptor co-repressor  Calcium binding tyrosine-  Calmegin  Calmodulin 2  Calmodulin 3  Calumenin  CAP, adenylate cyclase-associated protein 1  Capping protein  Capping protein  Carbonic anhydrase VI  Caspase 3  CBY1 interacting BAR domain containing 1  CCR4-NOT transcription complex, subunit 2  CD59 molecule, complement regulatory protein  CDC28 protein kinase regulatory subunit 2  CDC5 cell division cycle 5-like  CDGSH iron sulfur domain 1  Centrin 4  Centrin, EF-hand protein, 1  Centrosomal protein 57 kDa  Chaperonin containing TCP1, subunit 2  Chloride intracellular channel 4  Choline phosphotransferase 1  Cilia and flagella associated protein 95  Clusterin  Cofilin 2  Coiled-coil domain containing  Coiled-coil-helix-coiled-coil-helix domain containing 7  Coilin  Cold shock domain containing E1, RNA-binding  Cold shock domain protein A  CREB/ATF Bzip Transcription Factor  Cullin 3  Cyclin-dependent kinase-like 3  Cylicin, basic protein of sperm head cytoskeleton  Cysteine and glycine-rich protein 3  Cysteine dioxygenase, type I  Cysteine rich secretory protein 2  Cytochrome c oxidase subunit, mitochondrial  Cytokine induced apoptosis inhibitor 1  DCN1, defective in cullin neddylation 1, domain containing 1  DCN1, defective in cullin neddylation 1, domain containing 4  Defender against cell death 1  Diazepam binding inhibitor  Dickkopf-like 1  Dnaj heat shock protein family  DPY30 domain containing 1  DPY30 domain containing 2  Dynactin 6  Dynein light chain LC8-type 1  ELK4, ETS-domain protein  Enolase 1,  EP300 interacting inhibitor of differentiation 3  Eukaryotic translation elongation factor 1 alpha 1  Eukaryotic translation elongation factor 1 delta  Eukaryotic translation initiation factors  Eukaryotic translation termination factor 1  FANCD2 opposite strand  Fas apoptotic inhibitory molecule 2  Ferritin heavy chain 1  FGF receptor activating protein 1  Fibronectin type III domain containing 3A  Finkel-Biskis-Reilly murine sarcoma virus  FKBP prolyl isomerase 2  Fructose-bisphosphatase 1  FSHD region gene 1  Fumarate Hydratase  G protein subunit gamma 5  Galactokinase 1  Gametogenetin binding protein 2  GDP dissociation inhibitor 2  General transcription factor IIH, polypeptide 5  Germ cell associated 1  Glucose phosphate isomerase  Glutamate rich 2  Glutamate-ammonia ligase  Glutaredoxin 2  Glutathione peroxidase 4  Glutathione S-transferase mu 3  Glycosylphosphatidylinositol anchored molecule like  GNAS complex locus  Golgi associated RAB2 interactor 5A  Growth hormone inducible transmembrane protein  H2A histone family, member Z  H2B clustered histone 12 and 21  H3.3 histone A  Heat shock 70 kDa protein 5  Heat shock 90 kDa protein 1, beta  Heat shock factor binding protein 1  Heat shock protein 90 alpha family class A member 1  Heat shock transcription factor, Y-linked 2  Heterogeneous nuclear ribonucleoprotein C, D, and U  High mobility group nucleosomal binding domain 3  High-mobility group Box 1  Histidine triad nucleotide binding protein 1  Histone H2B variant PT15  HOATZ cilia and flagella associated protein  Homeobox A4  Huntingtin interacting protein K  Inflammation and lipid regulator with UBA-like and NBR1-like domains  Inhibitor of DNA binding 2, dominant negative helix-loop-helix protein  Insulin-like growth factor binding protein 7  Integrator complex subunit 12  Karyopherin alpha 4  Kelch like family member 10  Kelch-like 10  Kinesin Family Members  Kinesin heavy chain member 2A  Lactate dehydrogenase C  Leucine zipper protein 1  Leukemia NUP98 fusion partner 1  Lipid droplet associated hydrolase  Lysosomal protein transmembrane 4 alpha  Lysozyme-like 2  Male-enhanced antigen 1  Maternal embryonic leucine zipper kinase  Mediator complex subunit 31  Mediator complex subunit 6  Meiosis-specific nuclear structural 1  Membrane-spanning 4-domains, subfamily A, member 13  Metal response element binding transcription factor 2  Methyltransferase like 13  Mitochondrial fission regulator 1  Mitochondrial ribosomal protein S18C  Mitochondrial ribosomal protein S33  Mitochondrial ribosomal protein S9 |

**Table S2** Summary of differentially expressed transcripts in boar spermatozoa with normal and below-normal fertility

| **Highly expressed in boar spermatozoa with normal fertility** | **Highly expressed in boar spermatozoa with below-normal fertility** |
| --- | --- |
| 1-acylglycerol-3-phosphate O-acyltransferase 2  5-hydroxytryptamine receptor 4, G protein-coupled; 5-hydroxytryptamine receptor 4  Antioxidant 1 copper chaperone  ARP3 actin-related protein 3 homolog B (yeast)  ATP/GTP binding protein-like 5  Calcium regulated heat stable protein 1, 24 kDa  Casein kinase 1, gamma 2  Catsper channel auxiliary subunit gamma  Chromosome 1 open reading frame, human c9orf16; lipocalin 2  Chromosome 12 open reading frame, human c17orf97  Chromosome 9 open reading frame, human c11orf71  Coactivator-associated arginine methyltransferase 1  Coiled-coil-helix-coiled-coil-helix domain containing 5  Cyclin Y  Cytoplasmic polyadenylation element binding protein 3  DeSUMOylating isopeptidase 1  Dual specificity phosphatase 18  E3 ubiquitin-protein ligase NRDP1; E3 ubiquitin-protein ligase NRDP1-like  E3 ubiquitin-protein ligase RNF169  EH-domain containing 1  Eukaryotic translation initiation factor 4A3; eukaryotic initiation factor 4A-III-like  Family with sequence similarity 46, member C  Family with sequence similarity 57, member A  F-box and WD repeat domain containing 5  G protein-coupled receptor kinase 4  Glutathione peroxidase 4  Guanine nucleotide binding protein (G protein) alpha 12  Interferon stimulated exonuclease gene 20 kDa-like 2  KH domain containing, RNA binding, signal transduction associated 3; KH domain-containing, RNA-binding, signal transduction-associated protein 3-like  Maestro heat-like repeat-containing protein family member 1  Methenyltetrahydrofolate synthetase domain containing  Microfibrillar-associated protein 3-like  Microtubule-associated protein 1 light chain 3 alpha  Mitochondrial elongation factor 1  NADH dehydrogenase (ubiquinone) 1 alpha subcomplex, 10, 42 kDa  NADH:ubiquinone oxidoreductase core subunit S7  NADH:ubiquinone oxidoreductase subunit A10  NADH:ubiquinone oxidoreductase subunit V3  Neuralized E3 ubiquitin protein ligase 1  NOP2/Sun domain family, member 4  Nuclear transcription factor Y subunit alpha; nuclear transcription factor Y, alpha  Oxysterol binding protein 2  Peroxiredoxin 4  Phosphorylase kinase, gamma 2 (testis)  Pim-1 proto-oncogene, serine/threonine kinase  Pim-3 proto-oncogene, serine/threonine kinase; bromodomain containing 1  Protamine 2  Protamine 3  Proteasome inhibitor subunit 1; proteasome inhibitor PI31 subunit  Protein phosphatase 1, regulatory subunit 16A  Rab acceptor 1 (prenylated)  RAB7A, member RAS oncogene family  Receptor accessory protein 2  Replication factor C subunit 1-like  SAP domain containing ribonucleoprotein  Small G protein signaling modulator 2  SUMO1 activating enzyme subunit 1  Synaptonemal complex central element protein 2  Testis-specific serine kinase 2  Testis-specific serine kinase 6  Tetratricopeptide repeat domain 7A  TIMP metallopeptidase inhibitor 2  Transition protein 1  Transition protein 2  Transmembrane protein 239; chromosome 17 open reading frame, human c20orf141  TSSK6 activating co-chaperone  Ubiquilin-like  Ubiquitin-conjugating enzyme E2D 2  Vaccinia related kinase 3  Verprolin-like  Wingless-type MMTV integration site family, member 3 | ATP synthase subunit d, mitochondrial-like  E3 ubiquitin-protein ligase RNF220  Equatorin  Forkhead box P2; forkhead box protein P2-like  Interferon-delta-4; interferon tau-11-like  Olfactory receptor 1J4-like  Olfactory receptor 2AJ1-like  Olfactory receptor 4K1  Olfactory receptor 4K15  Olfactory receptor 52N4  Olfactory receptor 8H1-like  Porcine seminal protein I  Porcine seminal protein II  SLC9A3 regulator 1 |

Table S3 Summary of differentially expressed proteins in bull spermatozoa with normal and below-normal fertility

| **Highly expressed in bull spermatozoa with normal fertility** | **Highly expressed in bull spermatozoa with below-normal fertility** |
| --- | --- |
| Actin-like 7B  Acyl-CoA thioesterase 9  Adenylate kinase isoenzyme 1 (AK1)  A-kinase anchor protein 4  Albumin  Alpha-2-HS-glycoprotein  Apoptosis-stimulating of p53 protein 2  ATP synthase, H+ transporting, mitochondrial  ATP synthase, H+ transporting, mitochondrial F0  ATP synthase, H+ transporting, mitochondrial F1 complex  Casein kinase 2, alpha prime polypeptide  Cyclin-2  Cytochrome c oxidase subunit III  Diazepam-binding inhibitor-like 5  Enolase 1  Mitochondrial ATP synthase, O subunit  NADH dehydrogenase (ubiquinone) 1 alpha subcomplex  Ornithine aminotransferase, mitochondrial  Phosphatidylethanolamine-binding protein 1 (PEBP1)  Phospholipid hydroperoxide glutathione peroxidase, mitochondrial  Potassium voltage-gated channel shaker-related  Prohibitin  Rhabdoid tumor deletion region gene 1  Sperm associated antigen 6  Sperm mitochondria-associated cysteine-rich protein  Tektin 1  Tetraspanin-8  Transmembrane protein 5  Triosephosphate isomerase  Triosephosphate isomerase | Aldose reductase  Annexin A2  ATP synthase-coupling Factor 6, mitochondrial  Binder of SPerm-1  Clusterin  Dystonin-like isoform-1  Glutathione peroxidase  Glycoprotein (transmembrane) nmb  HADHA protein  Heat shock protein HSP 90-alpha  Heat shock-related 70 kDa protein 2  Isoform 2 of spermatid-associated protein  Lysyl oxidase-like 4  NADH dehydrogenase (ubiquinone) 1 beta subcomplex  Ropporin-1  Solute carrier family 25 (Carnitine/acylcarnitine translocase), member 20  T-complex protein 1 subunit alpha  Transmembrane protein-43  Voltage dependent anion channel 2 |

Table S4 Summary of differentially expressed proteins in boar spermatozoa with normal and below-normal fertility

| **Highly expressed in boar spermatozoa with normal fertility** | **Highly expressed in boar spermatozoa with below-normal fertility** |
| --- | --- |
| 1-phosphatidylinositol 4,5-bisphosphate phosphodiesterase delta-4  40s ribosomal protein sa  6-phosphogluconate dehydrogenase, decarboxylating (fragment)  78 kDa glucose-regulated protein  Acetyl-coenzyme a acyltransferase 2  Aconitate hydratase, mitochondrial  Acrosomal vesicle protein 1  Actin, cytoplasmic 1  Acyl carrier protein  Adenomatous polyposis coli 2  Adenosylhomocysteinase  ADP, ATP carrier protein isoform t2 (fragment)  ADP/ATP translocase 3  Adrenodoxin, mitochondrial  A-kinase (PRKA) anchor protein 3  Annexin  ATP synthase subunit delta, mitochondrial  ATP synthase subunit e, mitochondrial  ATP synthase subunit f, mitochondrial  ATP synthase subunit o, mitochondrial  ATP synthase-coupling factor 6, mitochondrial  ATPase inhibitor, mitochondrial  Calcium-binding tyrosine phosphorylation-regulated protein variant 1  Calmodulin  Cardiac muscle alpha actin 1  Cathepsin b  Chaperonin 10  Chromogranin-a  Cofilin-1  Cystatin-b  Cytochrome b-c1 complex subunit 2  Cytochrome b-c1 complex subunit 6  Cytochrome c  Cytochrome c oxidase subunit 5b, mitochondrial  Cytosolic 5-nucleotidase 1b  Dihydrolipoyllysine-residue succinyltransferase component of 2-oxoglutarate dehydrogenase complex, mitochondrial  Dj-1 protein  Electron transfer flavoprotein subunit beta  Elongation factor 1-alpha  Epidermal fatty acid-binding protein  Fumarate hydratase, mitochondrial  Glycerol-3-phosphate dehydrogenase  Haptoglobin  Heat shock protein beta-1  Histone h2a  Histone h2b  Histone h3  Histone h4  Hydroxyacyl-coenzyme a dehydrogenase, mitochondrial  Hypoxanthine-guanine phosphoribosyltransferase 1  Inositol monophosphatase 1  Inter-alpha-trypsin inhibitor heavy chain h4  Isocitrate dehydrogenase [nad] subunit beta, mitochondrial  Junction plakoglobin  Keratin 2a  L-amino-acid oxidase  L-lactate dehydrogenase  Lysozyme-like protein 4  Malate dehydrogenase  Micos complex subunit mic13  Mitochondria-eating protein  Mitochondrial malate dehydrogenase 2, nad  Mitochondrial nadh dehydrogenase Fe-S protein 4  Myelin basic protein  Na(+)/h(+) exchange regulatory cofactor nhe-rf  N-acetylglucosamine-6-sulfatase  Nadh dehydrogenase [ubiquinone] 1 alpha subcomplex subunit 2  Nadh-cytochrome b5 reductase  Nadh-cytochrome b5 reductase 3 (fragment)  Oxidative-stress responsive 1  Peroxiredoxin 2  Phosphatidylethanolamine-binding protein 4  Phosphoglycerate kinase 1  Phosphoglycerate mutase 2  Protein disulfide-isomerase  Pyruvate kinase (fragment)  Radixin  Ribonuclease  Saposin-b-val  Single-stranded DNA-binding protein  Sperm acrosome associated 4  Sperm acrosome associated 5  Sperm associated antigen 6  Spermadhesin psp-ii  Stress-70 protein, mitochondrial  Succinate dehydrogenase [ubiquinone] cytochrome b small subunit, mitochondrial  Superoxide dismutase  Testis anion transporter 1  Transcription factor a, mitochondrial  Trifunctional enzyme subunit alpha, mitochondrial (hadha)  Tubulin beta chain  Ump-cmp kinase  Vesicle-associated membrane protein-associated protein b  Wap four-disulfide core domain protein 2 | Spermadhesin aqn-3  Spermadhesin awn  Triosephosphate isomerase  60 kDa heat shock protein, mitochondrial (hspd1)  Acrosin-binding protein precursor (proacrosin-binding protein sp32)  Actin related protein t3  Actin-related protein t2  ADH dehydrogenase ubiquinone iron-sulfur protein 2  Alpha-mannosidase (fragment)  Angiogenin  Arginine vasopressin receptor 2  ATP synthase subunit d, mitochondrial  Beta-defensin  Beta-tubulin  Ceruloplasmin  Chain b, crystal structure of bovine mitochondria  Cytochrome b-c1 complex subunit 1  Cytochrome b-cl complex subunit 1, isoform2, mitochondrial  Cytoplasmic light-chain dynein  Cytosolic 5′ nucleotidase ib isoform 1  Equatorin  Glutathione peroxidase 4  Glutathione s-transferase mu3  Homo sapience cgi-104 protein mrna, complete cds  Hormone-sensitive lipase  Lipocalin 5 (fragment)  Low mr zona pellucida binding protein (fragment)  Monocarboxylic acid transporter 1 (fragment)  Mutant beta-actin (beta’-actin)  Nad(p)(+)--arginine ADP-ribosyltransferase (fragment)  Pancreatic glycoprotein 2  Pancreatic secretory granule membrane major glycoprotein (fragment)  Porin  Prohibitin  Pyruvate dehydrogenase: subunit = beta precursor  Ras-related protein rab-2a  Ras-related protein rab-2a  Seminal plasma protein pb1  Seminal plasma sperm motility inhibitor  Speriolin  Spermadhesin aqn-3  Syntaxin-binding protein 2  T-complex protein 1 subunit gamma (fragment)  Ubiquitin carboxyl-terminal hydrolase  Wap four-disulfide core domain 10a-like (fragment) |

Table S5 Summary of differentially expressed metabolites in bovine spermatozoa with normal and below-normal fertility

| **Highly expressed in bovine spermatozoa with normal fertility** | **Highly expressed in bovine spermatozoa with below-normal fertility** |
| --- | --- |
| Hypotaurine  Selenocystine  2′-Deoxyinosine triphosphate  l-Malic acid  Deoxyuridine triphosphate  Carbamoyl phosphate  Tg180/180/180  Iodide  Methacrylyl-CoA  N-Carbamoylputrescine  2,3-Diacetoxypropyl stearate  Glycerol tripropanoate  Glycerophosphocholine  l-Acetylcarnitine  gamma-aminobutyric acid  Lactic acid  Benzoic acid  Carbamate | d-Cysteine  β-d-Glucosyl-N-(docosanoyl)sphingosine  3-Mercaptolactic acid  Lactyl-CoA  Chondroitin 4-sulfate  Formyl-CoA  Acetoacetyl-CoA  FMNH2  3-Nitropropanoic acid  3-Phosphoadenylylselenate  Glycine Betaine  Butyrylcarnitine  l-Carnitine  Piperidine  Palmitic acid (C16:0) |
